# ‘She’s so knowledgeable about autism’: autistic women and birthing people’s experiences of specialist perinatal mental health teams

**DOI:** 10.1192/bjo.2026.12045

**Published:** 2026-07-14

**Authors:** Verity Westgate, Caitlin Thompson, Doretta Caramaschi, Heather A. O’Mahen

**Affiliations:** Department of Psychology, University of Exeter, UK; Population Health Sciences Institute, Newcastle University, UK

**Keywords:** Autistic spectrum disorders, perinatal psychiatry, mental health services, patients and service users, qualitative research

## Abstract

**Background:**

Autistic women and birthing people (AWBP) are at heightened risk of perinatal mental health difficulties such as perinatal anxiety and depression, yet little is known about their experiences of support from specialist perinatal mental health teams (SPMHTs).

**Aims:**

We aimed to understand the experiences of AWBP engaging with support from SPMHTs.

**Methods:**

We conducted semi-structured interviews with 18 participants from 4 SPMHTs in England to explore their experiences and perceptions of care. We used codebook thematic analysis to identify and develop themes that captured and explained patterns within the data.

**Results:**

We identified three themes: (a) seeing the whole person: for many, SPMHT involvement was the first time autism was recognised or explored in a mental health context. Participants valued opportunities for formal assessment, which supported self-understanding and highlighted the need for integrated care addressing both autistic and mental health needs. (b) Experiencing SPMHT care as an autistic person: participants valued care that accommodated their needs and preferences, particularly practical interventions, although several reported unmet needs and many found discharge challenging. (c) Ideas for service improvement: participants recommended access to autism-informed practitioners and peer groups tailored for AWBP.

**Conclusions:**

This study highlights how SPMHT care that recognises and responds to the distinct needs of AWBP can make a meaningful difference. AWBP valued holistic, autism-informed perinatal mental healthcare that recognised their communication, sensory and cognitive needs. Accessing assessment for a formal diagnosis supported the development of identity as an autistic person, helping to make sense of challenges in the perinatal period.

Many autistic women and birthing people (AWBP) experience mental health difficulties during the perinatal period (defined as pregnancy and the first year after giving birth), and are at greater risk of experiencing these than their non-autistic peers.^1–3^ This heightened vulnerability reflects the higher prevalence of mental health difficulties among autistic people, particularly those assigned female at birth,^4^ and their increased risk of suicide.^5^

Recent systematic reviews have identified a range of challenges faced by AWBP during the perinatal period.^6–9^ These challenges can be grouped into four key areas. First, many AWBP experience significant sensory difficulties, both during pregnancy and when caring for an infant. Second, healthcare environments are often inaccessible to AWBP, due to their sensory demands and the need to engage with professionals who may not understand autism or be able to tailor care appropriately. Third, parenting can place demands on executive functioning, which may be particularly difficult for some AWBP. Finally, the greater risk of experiencing perinatal mental health difficulties compared with non-autistic individuals can compromise executive functioning at a time of heavy adjustment and infant care demands.

Given these compound challenges, there is a lack of literature about the experiences of AWBP engaging with mental health services for support during the perinatal period. Specialist perinatal mental health teams (SPMHTs) have been implemented across the UK National Health Service (NHS) since a government investment of over £350 million in 2016.^10^ These provide multidisciplinary care, consisting of psychiatrists, community psychiatric nurses, psychologists and psychological therapists, social workers, nursery nurses and peer support workers, for women and birthing people with moderate to severe perinatal mental health difficulties. However, to date, only one study has explored the experiences of AWBP engaging with these services.^11^ The authors of that study interviewed five autistic patients accessing one service in England and found that participants valued individualised care, choice in appointment location and continuity with their lead practitioner. However, they reported that group-based online dialectical behaviour therapy was poorly received across the sample, and the study highlighted a need for greater autism training among staff to support early identification and appropriate care.

More broadly, research consistently shows that autistic people encounter obstacles when accessing and engaging with mental healthcare and that existing services do not adequately meet their needs.^12^ Autistic people encounter significant healthcare inequalities linked to barriers such as difficulties booking appointments by telephone, communication breakdowns with clinicians – explained by the ‘double-empathy problem’ (i.e. mutual misunderstanding between autistic and non-autistic people^13^), and sensory challenges in waiting room environments.^14^ Additional hurdles arise when healthcare professionals lack autism-specific knowledge or training, which can hinder recognition of needs and appropriate treatment planning.^15–17^ Access to psychological therapies is particularly affected by therapists’ knowledge of autism or reluctance to adapt approaches for autistic patients.^18^ For autistic women, barriers are compounded by the historical under-recognition of female-specific presentations, with symptoms often misinterpreted or overlooked,^19^ and by the use of camouflaging strategies, which women report more often.^20^

Taken together, the literature demonstrates both the heightened vulnerability of AWBP in the perinatal period and the substantial barriers they may face in engaging with healthcare. This study, therefore, explores the experiences of AWBP accessing SPMHTs, with the aim of informing service development and ensuring that perinatal mental healthcare better meets the needs of AWBP.

The research question is: how do AWBP experience engaging with SPMHTs?

## Method

We employed a qualitative descriptive design^21^ to capture in-depth accounts from AWBP about their experiences of engaging with care within SPMHTs. We recruited participants from four services in England with whom we had worked on an earlier study exploring staff experiences of working with autistic people in SPMHTs.^22^

Ethical approval was obtained from the West of Scotland 3 Research Ethics Committee (no. 24-WS-0064) and the South West–Central Bristol Research Ethics Committee (no. 19/SW/0218). The authors assert that all procedures contributing to this work comply with the ethical standards of the relevant national and institutional committees on human experimentation, and with the Helsinki Declaration of 1975 as revised in 2013.

This study was strengthened by an insider approach: V.W. is an autistic researcher with lived experience of SPMHT care following the birth of her daughter, and C.T. is an autistic autism researcher.

### Participants

Participants were eligible for inclusion if they met the following criteria:

- identified as autistic;
- had attended at least one appointment with a SPMHT;
- were nearing discharge from the SPMHT, to allow for reflection on their overall experiences within the service;
- had sufficient English proficiency to participate in a verbal interview or respond to questions via email.

We chose to include participants who lacked a formal diagnosis of autism but who self-identified as autistic, because diagnostic barriers often exclude marginalised groups.^23^

We recruited participants via their clinician (psychiatrist, care coordinator or occupational therapist), who provided an information sheet and explained that the lead researcher was an autistic woman with lived experience of care from a SPMHT. With participants’ consent, clinicians shared contact details with the research team, after which V.W. contacted individuals to provide further information, answer questions and arrange an interview.

Eighteen people took part in the interviews (see Table 1 for their characteristics).

**Table 1:** Participant characteristics

Characteristics	*N*
Autistic identity	
Formal diagnosis	12
Self-identified	6
Ethnicity	
White British	16
Black and minority ethnic British, or not given	2
Number of children	
1	9
2 or more	9
Point of referral	
Antenatal	10
Postpartum before 6 weeks	6
Postpartum after 6 weeks	2
Mental health and other neurodivergent diagnoses	
Anxiety	9
Depression	7
Emotionally unstable personality disorder	5
Attention-deficit hyperactivity disorder	5
Post-traumatic stress disorder	2
Birth trauma	2
Bipolar disorder	1
Age of child at discharge, months	
<6	1
6–12	14
12+	3

### Procedure

We carried out interviews between March 2024 and June 2025. Participants provided written or audio-recorded informed consent. Participants were told that (a) confidentiality would be broken only in the event of a safeguarding disclosure, (b) they could request a break at any time and (c) they were not obliged to discuss any topic they did not wish to. We provided participants with a summary of topics to be discussed in advance.

We conducted most interviews via Microsoft Teams, with adjustments as preferred, including Teams with the camera off (six participants), telephone interviews (one participant) and email responses (one participant). All participants received a £20 thank-you shopping voucher.

V.W. led all interviews using a semi-structured topic guide. Interviews ranged from 13 to 71 min, with a median of 44 min. Interviews were audio-recorded, transcribed, pseudonymised and assigned a unique identifier. V.W. completed a reflective diary after each interview.

### Data analysis

Data were uploaded to NVivo (version 14 for Windows, Lumivero, Denver, CO, USA; see https://lumivero.com). We used codebook thematic analysis, which is a structured form of thematic analysis that uses a clearly defined coding framework developed by the research team, to organise and interpret the data. It is a method particularly suited to collaborative and applied research contexts, aligning with our broader applied aims to collaboratively inform health services.^24^

V.W. familiarised herself with the data through repeated reading of transcripts. Coding began after four interviews, with inductive codes generated through close attention to participants’ accounts. Early codes were discussed with H.A.O., and the codebook was refined as further interviews were analysed. C.T. independently reviewed and coded 15% of the transcripts, providing interpretive feedback that informed the codebook. V.W. developed preliminary themes and subthemes, which were then discussed with the wider team. We refined themes against the full data-set, and all authors agreed on the final themes.

## Results

**Fig. 1:**
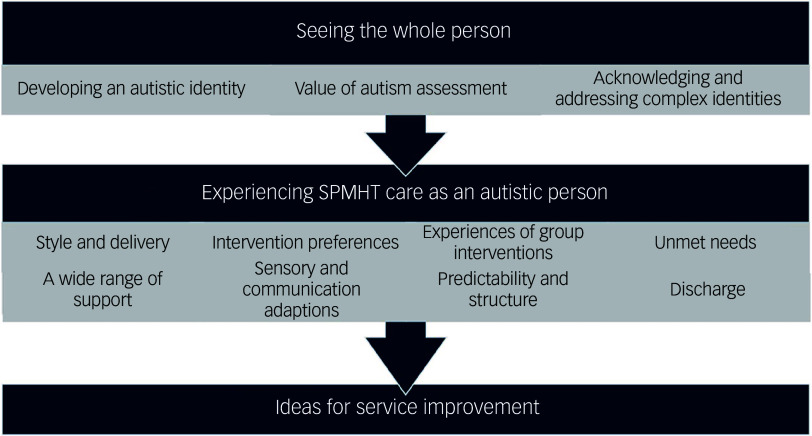
Overview of themes identified. SPMHT, specialist perinatal mental health team.

The themes identified are illustrated in Fig. 1.

### Seeing the whole person

Only three women received a formal autism diagnosis before accessing the SPMHT, and many described their engagement with the service as the first time that autism was recognised. This recognition helped them understand themselves in new and often profound ways.

#### Developing an autistic identity

Developing an autistic identity was often a path of surprise and adaptation. For several participants, autism had not been considered before: ‘she said to me then, have you ever thought you might be autistic? And I went, no’ (Participant 14). This participant, like many in the study, ultimately found that the presentation fitted them well. Perinatal stresses often made the signs of autism more apparent:

‘my OT [occupational therapist] uses the cup analogy… I had so much in my cup before and when things got added I could manage it, whereas now my cup is already nearly full because of the children and everything that when these other things go in it just overflows a lot more easily’ (Participant 14).

A number of women who had not previously been recognised or identified as autistic reflected on the alternative diagnoses they had received: ‘I had seen and spoken to many doctors or therapists before and the connection had never been made’ (Participant 13). Notably, these other diagnoses frequently did not resonate with participants’ own experiences. Of the alternative diagnoses, personality disorder was most common:

‘my own GP referred me as an adult for mental health diagnosis. And they diagnosed me with a personality disorder. And from that moment… I was like, but I don’t have a personality disorder’ (Participant 8).

For many women, having autism recognised, often for the first time, was a turning point. It provided a clearer, more compassionate understanding of themselves and replaced previous misdiagnoses with an identity that finally made sense.

#### Value of autism assessment

Women who had not received a formal diagnosis prior to SPMHT involvement appreciated the offer of a formal assessment, because it helped them gain clarity about the characteristics influencing their experiences. Given long wait times in the NHS for formal autism assessments through autism services, two services offered in-house assessments, enabling six participants to be diagnosed during the perinatal period. Two other participants received assessments from NHS autism services, with one fast-tracked to support understanding of her current situation: ‘they put my referral down as priority… So, the entire start to end process happened whilst I was under perinatal’ (Participant 3). This participant described her diagnosis as ‘clarifying’.

However, in a small minority, assessment and diagnosis were not always immediately welcome. It took some women time to adapt to the idea of potentially being autistic. One participant reflected: ‘immediately when she told me, I was like, I can’t handle this because I was deep in the throes of my postnatal depression at the time’ (Participant 12). As her mental health improved, however, she later came to view the diagnosis as helpful: ‘with the following appointment… I was… getting better at that point with my depression. I was then like, oh, yeah, this makes sense’.

Not all participants received a formal diagnosis while under a SPMHT because not all services could provide assessments, but hearing a professional validate their experiences was helpful: ‘So, yeah, having somebody actually say “Yeah, no, it’s neurodivergence and it’s the way your brain works“, it was like, yeah, it was helpful’ (Participant 16).

#### Acknowledging and addressing complex identities

Being seen and understood holistically was important for all of the AWBP, and they strongly preferred support that addressed the interplay between being autistic and mental health, which they saw as closely linked: ‘I didn’t just come along with autism. I’ve got PTSD [post-traumatic stress disorder], borderline personality disorder, I’ve got… ADHD [attention-deficit hyperactivity disorder] um and um there’s another diagnosis’ (Participant 4). SPMHTs, however, did not always provide care that addressed these complex interrelationships. Several participants reported that SPMHT care focused more on one need to the detriment of the other:

‘what I found is they’ll either focus on me being autistic or they’ll focus on me being borderline. It’s never a borderline autistic… I’m not… different people I am one person. This is one thing. They do all interact with each other’ (Participant 3).

One participant was assessed by the SPMHT but referred back to treatment in primary care mental health NHS Talking Therapies because, although they recognised that she was autistic, her mental health needs were not considered sufficiently complex for specialist support. However, the NHS Talking Therapies service focused primarily on mental health problems, rather than on neurodivergence or its interplay with mental health. She noted that the SPMHT clinician acknowledged a gap in provision for neurodivergent people:

‘they told me… they recognise that there is a gap in their services for neurodivergent people who are struggling not so much with… a consistent depression or a consistent anxiety just struggling with those changes [from having a new baby]’ (Participant 1).

The participants valued staff who had a strong understanding of autism and its relationship to their mental health needs; this enabled professionals to respond more effectively to the women’s complex needs across the perinatal period: ‘She’s so knowledgeable about autism in women and mums that it’s been… a great support’ (Participant 14). It was also valuable to receive emotional validation that recognised that being autistic might make things particularly challenging: ‘She’s really helping me find all the tips and tricks and just like telling me that, you know, it’s okay to have an overwhelming day’ (Participant 10). They appreciated those who understood their way of being: ‘She… didn’t mind me fidgeting a lot… I feel like she was really patient with me as well which was really helpful’ (Participant 9).

However, participants recognised that staff did not always have the necessary knowledge and therefore respected those who were willing to learn from them:

‘she’s definitely very good at kind of asking me questions. What I need and why I need something which is really helpful… I think she’s really grateful that I can give her a perfect insight into what it’s like to be an autistic woman’ (Participant 6).

### Experiencing SPMHT care as an autistic person

Most participants expressed strong appreciation for the care they received from the SPMHT, particularly for being offered consistent support. Nevertheless, experiences of care were variable and depended on the extent to which staff understood individual autism profiles and characteristics.

#### A wide range of support

Participants reported a wide range of interventions and support received (Table 2).

**Table 2:**
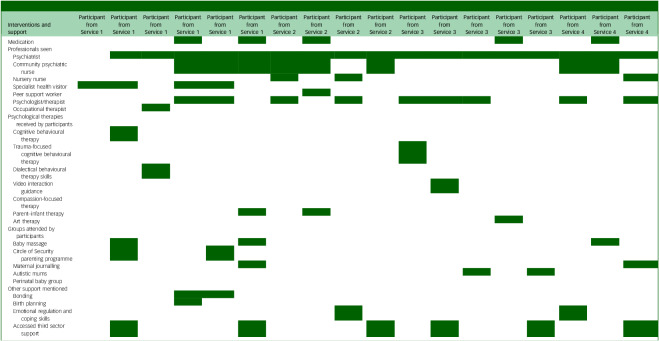
Interventions and support received by participants

Service 1–4 denotes which service a participant accessed; the shaded boxes show which interventions they recieved.

Care provision varied among sites and participants, although almost all saw a psychiatrist and nearly half a community psychiatric nurse. There was greater variability in what participants received within a service than between services. A minority of participants described receiving two or three interventions, and some engaged in active, shared decision-making about which types of support they wanted. These participants expressed high levels of satisfaction with their care: ‘all these things were open to me and I just went for them all and I’m… glad I did… I’ve definitely learned stuff from all of the elements’ (Participant 12). Another participant described it as ‘probably the best mental health support I have had’ (Participant 3), but this may have reflected the contrast with the lack of understanding and limited therapeutic offer that person had experienced in general mental health services.

#### Intervention preferences

Participants discussed clear preferences regarding the types of interventions they found most accessible and effective. They particularly valued practical, concrete interventions because these did not rely on abstraction or the ability to label emotions, and could provide something tangible to work on. Video interaction guidance was described as helpful because it used video to demonstrate specific details in the interaction in the parent–child relationship and to use those to guide future behaviours: ‘you can see the bit-by-bit all the tiny little human interaction that you don’t even think about’ (Participant 12). On the other hand, alexithymia could make therapeutic approaches based on feelings challenging: ‘We had CBT [cognitive–behavioural therapy] and everything, but CBT doesn’t work… It doesn’t work on my brain… [because it is] based on being able to access your feelings and identify them’ (Participant 2). Similarly, it was tricky for some participants to respond to the standardised questionnaires often used by SPMHTs to measure progress:

‘I can’t answer half of those questions. Like, do you feel close to your baby? And I’m like, well, she’s always with me, so yes. But then they’re like, but do you feel, like, emotionally close? And I’m like, well… I don’t understand’ (Participant 5).

Occupational therapy was particularly helpful for this practical focus, especially when helping with sensory regulation: ‘We’ve done the sensory ladder and… a little box thing… to like try help with the things that dysregulate [me]’ (Participant 17).

#### Experiences of group Interventions

Participants described varied experiences of therapeutic groups, reflecting differing needs and preferences. Some participants found therapeutic groups positive, leading to beneficial interactions with peers. One participant described taking part in the Circle of Security intervention: ‘it’s a small group. And they don’t force you to interact, but they encourage it. As you start interacting… you learn a little bit about other people and things’ (Participant 5). In one service, some participants accessed a dedicated group for autistic women in the perinatal period. One attendee valued hearing others’ experiences, noting that it was ‘weirdly illuminating’ (Participant 15) because it helped her shift from feeling that something was wrong with her to recognising that her environment made things difficult. For her, validation of her experiences mattered more than connection with others in the group.

Some participants indicated that the therapeutic groups offered did not meet their current needs, often because of the social interaction required: ‘I find it difficult to meet [others]… it was a no-no, so that’s how I ended up sort of probably [with] more home visits than most’ (Participant 4). However, many participants whose services did not run a therapeutic group suggested that SPMHTs should offer a dedicated group for autistic people in the perinatal period. ‘To have… groups that we could go to on a regular basis and meet other mums that were kind of in a similar situation’ (Participant 6).

#### Unmet needs

Some participants explicitly identified support needs that remained unmet, often around getting out and about:

‘we were identifying what I needed, but I wasn’t getting what I needed. So, like, we’d identified that I needed birth trauma [support], and I’ve not had that. We identified that I needed some help with actually leaving my house, and I’ve not had that’ (Participant 3).

In other cases, participants described challenges that suggested unmet needs, even if they did not label them as such. One spoke about difficulties with leaving the house: ‘I think it was hard getting out anyway because of the post-traumatic stress and certain things would set me off’ (Participant 11), yet had not been offered any targeted support to address these issues. Only one of the four participants who reported experiences of trauma had received any kind of trauma therapy.

#### Style of delivery

Participants expressed their opinion that appointments were most effective when delivered in ways that matched their preferences and needs. For the majority, this meant face-to-face, home-based appointments: ‘I was really glad it was a home visit, because I was finding…going out of the house… really difficult’ (Participant 16). However, this was not always the case: ‘I prefer to meet them out in public because I feel like it’s easier for me to talk to them. But it has to be a place I’ve gone to before’ (Participant 9). Some might prioritise face-to-face appointments even if inconvenient: ‘I really preferred being seen in person… But it did mean having to drive like an hour each time, which was a lot when I had a young baby’ (Participant 6).

One participant forced herself to interact online as it was the only option offered: ‘if it’s the only way it’s provided, I know it’s going to really benefit me. So I’ve just braved it’ (Participant 5). Others found online preferable: ‘I know I’m in the comfort of my own home. I can go to the things that give me comfort instantly’ (Participant 11). However, telephone appointments were described as ‘stressful as you didn’t always know when you would get the call and so couldn’t mentally prepare’ (Participant 13).

#### Sensory and communication adaptations

Participants highlighted the importance of adapting appointments to support communication and engagement. Within the appointment, adaptions to facilitate communication were important. One participant explained: ‘I find like looking at giving people eye contact really difficult and stressful… they make sure that I don’t have to… sit directly facing them’ (Participant 8). Another described requesting some tools to help her engagement:

‘I find it really hard… sitting one-to-one talking with somebody without… anything to do. So I asked them… could you get some colouring pens? Could you get something that like can kind of distract me and occupy my hands whilst I’m talking to you?’ (Participant 6).

One participant emphasised the need for information to be communicated in different ways to aid processing and memory: ‘[Nothing] was ever followed up in writing… I struggle with verbal, and… I tend to forget… because I haven’t processed the information from the meeting’ (Participant 3).

Staff, particularly occupational therapists, could successfully incorporate sensory ideas into their practice. One occupational therapist helped a participant to make an ‘olive oil and hard salt rub’ (Participant 10) as a valued alternative to self-harm. Another participant found that an occupational therapist was able to help her with strategies to ‘manage things like sensory overload, which… was causing me a lot of difficulty in day-to-day life’ (Participant 13).

#### Predictability and structure

Successful care incorporated preferences for predictability and structure, particularly around appointment schedules. One participant said: ‘[I] like to know things in advance… and she kept me up to date. Like she said that she was leaving towards the end, but I still may need a couple of more sessions’ (Participant 11).

Others wanted the chance to have an appointment structure that suited them, particularly in terms of duration. Shorter sessions were described as valuable for a person who also had ADHD: ‘we normally only have… half-hour appointments… I don’t mean to sound rude when I say it, but I get bored’ (Participant 10). Yet for another, longer appointments were valued for allowing more time to engage meaningfully: ‘So you’ve got a chance to sort of talk, but… you haven’t got the clock ticking against you’ (Participant 4).

Structure was also particularly important when it came to discharge. This was experienced more positively when it was gradually implemented:

‘We’ve put a plan in place… I was seeing her weekly, and then we’ll drop to two-weekly, and then we’ll drop to monthly so that then I’m kind of getting used to that kind of dealing with things on my own’ (Participant 6).

Interventions that helped new parents establish predictability and structure amid executive functioning challenges were valued. One participant described the value of her occupational therapist assisting ‘with routines and organisational things… we’re talking about ways to… manage doing the packed lunch for my older daughter or helping her get ready for school’ (Participant 14).

#### Discharge

Discharge from the SPMHT was often challenging for many participants, particularly because they could struggle with transitions: ‘it feels quite at times overwhelming like you’ve kind of been monitored and kind of been offered this support for say 2 years and then all of a sudden it’s kind of like dropping off a bit of a cliff’ (Participant 6). More than half of the participants reported being discharged without any ongoing support because there were no NHS-funded services available to support them: ‘I don’t think there’s going to be anything to offer me’ (Participant 17).

### Ideas for service improvement

Participants were asked to reflect on how SPMHTs could be developed to better meet the needs of autistic patients. This task was occasionally difficult, because some were uncertain about the remit of SPMHTs. Participants wanted to be able to access practitioners to have a comprehensive understanding of autism: ‘the nurses should be trained up on… autism and not just autism as in one spectrum… across the board… you know how different people react’ (Participant 4). A related suggestion was for services to have an autism practitioner: ‘it would be really useful to just have somebody that could kind of be aware of what needs might come up’ (Participant 6).

Other participants reflected on aspects that would have enhanced their care, and recommended these for future service provision. One participant suggested a targeted antenatal course for AWBP: ‘[about] things you might encounter as an autistic parent and… things you can do to help cope’ (Participant 1). Practical support was often mentioned, especially relating to information processing and communication: ‘it could be just sitting and going through some letters… because it’s really hard to navigate stuff as a mum and being autistic’ (Participant 18). One participant described the need for better links between services: ‘so you’re not repeating yourself, or they’re not missing any of your needs’ (Participant 4).

## Discussion

Our study offers the largest account to date of the experiences of AWBP who have received care from a SPMHT. We found that, for many participants, engagement with SPMHTs represented the first time that autism was recognised or meaningfully explored in a mental health context, often leading to the development of an autistic identity that replaced earlier misdiagnoses, providing greater self-understanding. We found that participants benefitted from holistic care that recognised and responded to both their autistic needs and mental health challenges; they valued practical, concrete interventions alongside flexible and predictable modes of delivery. The findings suggest that having choice and a sense of control over care through shared decision-making and support aligned with individual preferences may be a key factor underpinning positive service experiences. However, unmet needs were common, most notably around trauma-focused support, getting out and about and discharge planning.

Our study adds to a growing literature highlighting systemic challenges in mental health service provision for autistic people, including barriers to access, limited understanding of co-occurring mental health needs and the lack of dedicated autism-informed pathways.^15^ This is emphasised by a 2023 report (Meeting the needs of autistic adults in mental health services^25^), which notes that autistic adults in England experience disproportionately high rates of mental health difficulties, requiring equitable access to services.

For many participants, gaining certainty about their autistic identity and having the opportunity for formal assessment during the perinatal period were vital in developing self-understanding, supporting previous findings that formal autism diagnosis can lead to self-acceptance^26^ and self-compassion.^27^ An assessment could also help someone understand how autism affects their new role as a parent and identify any necessary adaptations, navigating healthcare services or parenting, particularly because sensory experiences and planning and organisation can be challenging for autistic parents.^28^ A number of the participants had prior contact with mental health services and had not been identified as autistic, with other diagnoses such as personality disorder being given instead. This finding echoes broader research documenting the tendency for autism, particularly in those assigned female at birth, to be misidentified as a personality disorder^29^ or not recognised as being comorbid with personality disorder.^30^ However, access to autism assessment varied across services, with reliance on external pathways often leading to delays. This highlights the need for more joined-up services to support timely identification and care for autistic people in the perinatal period.

Participants’ care experiences were strongly influenced by their individual autistic characteristics, including communication preferences, sensory sensitivities, need for predictability and cognitive style. When care was adapted in these areas, participants were more likely to describe their support as good, but this depended on staff having a good understanding of autism and the implementation of reasonable adjustments for AWBP in the perinatal context. Many adaptations were relatively straightforward, consistent with findings from a systematic review around the importance of autism awareness, environmental and communication adjustments and individually tailored approaches in mental healthcare.^31^ Many of these are not only beneficial but are required under the UK Equality Act 2010,^32^ which places a legal duty on services to make reasonable adjustments to ensure equitable access for autistic people.

Some participants, however, reported experiences suggesting unmet needs. Notably, only one participant reported receiving treatment for PTSD despite several indicating experiencing symptoms, although it was not clear why this was the case in our study. Autistic adults may be more susceptible to PTSD^33,34^ and can face difficulties in having it identified due to the overlap with autistic traits and consequent diagnostic overshadowing,^35^ so it is important that SPMHT recognise and treat PTSD symptoms.

Additionally, challenges often arose around discharge from SPMHT care. Transitions can be testing for autistic people, who tend to benefit from routine and predictability, and participants frequently reported that there was no appropriate service for referral, even when support needs remained. Although there is little research specifically examining discharge experiences from community mental health services, Maloret et al^36^ have highlighted the heightened anxiety that autistic patients can experience when discharged from mental health hospitals. SPMHTs could consider planning discharge from an early stage, gradually and collaboratively, explicitly acknowledging when onward support is limited.

More positively, participants who joined a group specifically for AWBP valued it, and several who had not had the chance said they would have liked to attend one. This reflects the findings of Hampton et al,^2^ where the small number who accessed support from other autistic parents found it helpful, and many who hadn’t would have liked to. Beyond this population, peer support groups positively impact autistic people, who value connecting with others with similar experiences.^37^

Our study provides one of the first accounts of AWBP’s experiences of engaging with SPMHTs. Participants valued holistic, autism-informed approaches that addressed both their autistic and mental health needs. Services could consider ways to facilitate autism assessments in the perinatal period to help AWBP understand and frame their perinatal experiences, as well as ways to adapt care to meet communication preferences, sensory sensitivities, need for predictability and structure, and cognitive style. Peer support for AWBP is another potential area for future research and policy/practice development.

### Strengths and limitations

The study was informed by an insider perspective, with the lead researcher (V.W.) being an autistic woman with lived experience of accessing SPMHT care. This probably enhanced rapport with participants, some of whom felt anxious about taking part, and supported the depth and openness of the data collected. Her lived experience enriched interpretation; reflexivity was maintained through a reflective diary and regular discussions with the co-authors, enabling critical examination of assumptions and potential biases from her own individual experience, and the incorporation of multiple perspectives.

Although included participants represented a wide range of experiences of care, all but one were White British. This may reflect the demographic make-up of the areas where the four SPMHTs were based, as well as lower access of SPMHTs by Black and minority ethnic patients. However, people from Black and minority ethnic backgrounds are consistently underrepresented in autism research, underscoring the importance of identifying and implementing strategies to include them.^38^

This study was conducted in England, where specialist perinatal mental healthcare is delivered through multidisciplinary teams. Although this service context may limit transferability, comparable out-patient perinatal mental health services operate in countries including Ireland, Germany and the USA.^39^ The findings highlight the importance of care specifically designed for the perinatal period and informed by an understanding of autism, with autism and mental health needs addressed together rather than separately.

## Data Availability

The data that support the findings of this study are not openly available due to reasons of confidentiality, but are available from the corresponding author upon reasonable request.
